# The Determinants of Organic Vegetable Purchasing in Jabodetabek Region, Indonesia

**DOI:** 10.3390/foods5040085

**Published:** 2016-12-07

**Authors:** Alim Setiawan Slamet, Akira Nakayasu, Hu Bai

**Affiliations:** 1 The United Graduate School of Agricultural Science, Ehime University, Matsuyama 790-8566, Japan; 2 Department of Management, Faculty of Economic and Management, Bogor Agricultural University, Bogor 16680, Indonesia; 3 Department of Resource and Environmental Policy, Faculty of Agriculture, Ehime University, Matsuyama 790-8566, Japan; nakayasu@agr.ehime-u.ac.jp (A.N.); hu.bai.mm@ehime-u.ac.jp (H.B.)

**Keywords:** consumers’ purchase behavior, organic vegetable, binary logit model, Indonesia

## Abstract

Over the last few years, the global market of organic vegetables has grown. This is due to increased consumer concern regarding environmental and health issues, especially for food products. This study aims to examine factors that influence consumer behavior in purchasing organic vegetables. In this study, data were obtained from household surveys conducted in the Jabodetabek region (Greater Jakarta) from February to March 2015. Descriptive analysis, factor analysis, and a binary logit model were used to analyze the data. Subsequently, the results show that consumers with fewer family members and have a higher income, and are price tolerant, are more likely to purchase organic vegetables. Meanwhile, female consumers are less likely to buy organic vegetables. Another important finding is that positive attitude towards organic products, safety and health, environmental concerns, as well as degree of trust in organic attributes, are the determinants of organic vegetable purchasing among consumers. Therefore, based on the study results, the following recommendations are needed for organic vegetable development in Indonesia: (a) implementing an appropriate pricing strategy; (b) encouraging organic labeling and certification for vegetables; and (c) intensively promoting organic food with respect to consumers’ motives and concerns on health, safety, as well as environmental sustainability.

## 1. Introduction

The development of organic agriculture in Indonesia can be traced to the early 1980s as an alternative solution for the green revolution. The green revolution policy eventually led to several negative impacts, mainly on the environment which is caused by the excessive use of chemical inputs in order to achieve food self-sufficiency. The development of organic agriculture is meant to avoid this impact [1]. According to the latest data from the Research Institute of Organic Agriculture (FiBL) and the International Federation of Organic Agriculture Movements (IFOAM-Organics International) [2], the total area of organic agricultural land in Indonesia was 113,638 hectares in 2014, or the fourth largest organic area in Asia, after China, India, and Kazakhstan. The total area of organic agricultural land is equal to 0.2% with respect to the total area of agricultural land in Indonesia. Of this total organic area, only 67,426 hectares were certified as organic agricultural land, with 1142 hectares undergoing a certification process, and cultivated by 5700 farmers [1,2].

Although there are no reliable statistical data on organic food, its demands, both for exported and domestic markets, are increasing, and this trend is expected to continue [1]. Organic tea, coffee, and spices are important commodities that are mostly exported to Europe and the United States. Meanwhile, organic vegetables and organic rice are the most-consumed organic products by Indonesian consumers [1,3]. According to Indonesian Organic Alliance (AOI) [4], the domestic market share of organic food is dominated by fruits and vegetables (21%), followed by rice (20%) and honey (10%). The expansion of the domestic organic food market is driven by rising incomes, an increased level of education, changes in demographic structure, and urbanization. Income per capita of Indonesians has increased from 2324.48 USD in 2011 to 3227.14 USD in 2015 [5]. In addition, a report by the McKinsey Global Institute [6] indicates that Indonesia’s consuming class was 1.6 million in 2004, 45 million in 2010, and is projected to reach 135 million by 2030. Moreover, Indonesia will experience a demographic bonus, or demographic dividend, which is predicted to occur in 2025–2035. This will happen when the age structure is characterized by a high proportion of people in productive ages compared to non-productive ages (elderly people and children). Those changes will also create an inherent advantage by producing higher levels of per capita income [7,8]. In terms of education level, higher education enrollment rates reflect an impressive growth, from 17.26% in 2005 to 31.1% in 2014 [9]. Indonesia has also been facing a high urbanization rate, as the people living in urban areas has increased significantly, from 31% in 1990 to 53% in 2014, and is projected to increase to over 71% by 2050 [10]. The development of the domestic organic food market is also reinforced by changing lifestyles and diets as a result of campaigns by the government and others on the importance of healthy foods [1]. The rapid growth of the modern market in Indonesia has also accelerated the expansion of organic food markets. Currently, there are two types of organic marketing channels, i.e., mainstream supermarkets and specialized organic stores, that only sell organic products [11].

Consumer choices and behaviors on purchasing organic foods in Indonesia have been investigated in several studies Here, we review papers reporting empirical studies conducted in Indonesian that are available in English. Suprato and Wijaya [12] found that a healthy consumption lifestyle is a good predictor of attitude towards organic foods. Meanwhile, attitude towards organic foods directly influences the purchasing intention of organic foods. Similar results were also found in Reference [13]. This study suggests that consumers’ main reasons for choosing an organic products are health factors, vitamin content, dietary purpose, a product’s being satisfying, and to support the government’s “Go Green” program. Kurnia et al. [14] examined consumer perceptions of organic food in Yogyakarta. They found that socio-demographic variables, such as age, income, and education levels influence people’s perceptions towards organic food attributes. They also suggested that major factors preventing people from purchasing organic foods were a high price, a lack of availability, a lack of credentials, and a poor appearance. Wahida et al. [15] explored consumer willingness to pay for high-value agricultural products in Surabaya, Bogor, and Surakarta. Female respondents with children and living at home, those who were older, more educated, and more concerned about nutrition, and those indicating that they had previously purchased organic products were likely willing to pay more for certified organic food products. Their research results also suggested a further growth of demand for certified organic food products in Indonesia. Effendi et al. [16] studied consumer behavior with respect to organic foods in North Sumatera. Based on the expanded rational expectation model, a development of a model of theory of reasoned action, they found that consumer intention to buy organic foods was influenced by attitudes toward the organic foods and subjective norm variables. Specifically, they also found that health and organic food knowledge are important variables in shaping the attitudes towards organic products; meanwhile, environmental knowledge, organic food attributes, and cultural variables (consumer lifestyle) do not significantly influence attitudes toward organic foods.

From previous studies [12,13,14,15,16], Indonesian consumer choices with respect to organic foods seem to only be analyzed based on socio-demographics (e.g., age, education, income, and children in the household) and health reasons. Meanwhile, consumer preferences towards organic food are complex; not only linking food to health, but also linking food to their awareness of the environment, attitudes toward organic products, and trustworthiness of information [17,18,19,20,21]. This study attempts to provide a better understanding of consumer preferences on organic food markets in Indonesia. More specifically, the objectives of this paper are: (1) to explore the factors that drive Indonesian consumer preferences in the purchasing of organic vegetables, and (2) to determine the factors that help explain consumer preferences in the purchasing of organic vegetables. The paper is structured as follows. Section 2 covers the conceptual framework of this research. Section 3 describes the data and methods. In Section 4, we present the results in three parts: (a) descriptive information about sample characteristics; (b) factors driving organic vegetable purchasing; and (c) an estimation of the determinants. Section 5 features a discussion on the results, and Section 6 provides the conclusions and limitations of this study.

## 2. Conceptual Framework

Over the last two decades, various studies on organic consumers have been conducted in some countries in the EU and in other Western countries, such as the United States of America, Australia, and New Zealand, in order to examine organic food consumption and its determinants [18,22,23,24,25,26,27,28,29]. Despite a seemingly increased demand for organic food in Southeast Asian countries, there are only limited studies on organic marketing in this region (e.g., Thailand, the Philippines, Vietnam, Malaysia, and Indonesia) that present data on consumer preferences towards organic foods [19,21,30,31]. To describe the variables that determine consumer preferences for organic vegetable purchasing, we refer to previous research on organic consumers. Roitner-Schobesberger et al. [19] provided information on the preferences and the reasons why consumers purchase or do not purchase organic vegetables in Bangkok, Thailand. Organic buyers described tend to be older, have a higher education level, and have a higher family income relative to non-organic buyers. They found that the three main motivators to purchase organic vegetables are the expected health and environmental benefits, the attraction of new and fashionable products, and the search for tastier products. Similarly, health and environmental benefits were also found as the main consumer motivators to purchase organic foods in Northern Thailand [32]. In further research, Pomsanam et al. [21] demonstrated that the motives of Thai consumers’ intentions to purchase organic foods include subjective norms, environmental protection, trust in label, food quality, availability, and the convenience of accessing organic foods. Ara [33] revealed that organic attributes associated with reduced health risk level, organic certification, and environmental concern were the primary reasons for consumers in the Philippines to purchase organic rice. In Vietnam, health and safety motives positively influence potential consumer willingness to purchase organic foods, yet environmental and sustainability concerns did not affect purchasing decisions [34]. Hai et al. [35] found that the most important factors that influence the willingness to pay (WTP) of Vietnamese consumers are concerns (of price or safety and quality), income, and experience consuming organic products. In addition, Kai et al. [36] identified the determinants of the WTP for organic products in Klang Valley, Malaysia. These determinants include environmental awareness, health, perceived expensiveness, as well as labeling and certification, which significantly influenced consumers’ willingness to pay.

From the several motivators mentioned above, most previous studies found health and safety concerns to be the primary reason consumers buy organic foods [21,25,37,38,39,40]. Albeit there has been no solid evidence that organically produced foods are actually healthier than conventionally produced foods [41], perceived healthiness is the most frequently mentioned reason for many consumers for selecting organic foods [42]. Consumers buy organic foods because they believe that organic food is more nutritious and there is an inclination to avoid the chemicals used in conventional food production [31,42,43,44]. According to Guilabert and Wood [45], health and safety-related attributes (fewer chemical, fewer pesticides, naturally produced) are the consumer values and motivations for organic food purchasing behavior. Fear over harmful microorganism, such as salmonella and *Escherichia coli 0157* outbreaks, have also contributed to increasing consumer concerns regarding conventional food production methods [46].

In several studies, it has been found that attitudes towards organic foods are determinants of behavior and organic purchasing preferences [3,25,47,48,49]. The higher quality and better taste offered by organic foods are the primary reasons consumers buy them [43,50,51]. Krystallis et al. [18] found that most consumers strongly agree with these reasons to buy organic foods, i.e., healthier, superior in quality, and tastier. Similarly, Ergin and Ozsacmaci [52] assessed Turkish consumer attitudes and preferences towards organic food products. Results of their research indicate that the main reasons for purchasing organic foods are that consumers believe them to be healthier, tastier, and fresher. The higher price of organic foods also influence the perception of consumers, which consider organic foods to be of higher quality as compared to conventionally grown food [44]. Roitner-Schobesberger et al. [19] also noted that consumers’ reasons for purchasing organic products in Thailand was because they are fresher than other products.

Consumer motives regarding the environment have also been identified as a reason for the purchase of organically produced food [19,47,53,54,55]. Organically produced foods, due to the absence of chemicals and pesticides, are perceived as being better for the environment than conventionally grown food [43]. According to Nejadkoorki [56], there are several environmental benefits of organic farming, such as preserving biodiversity; reducing air pollution, not just from the lower carbon footprint, but also from the absence of chemical sprays that enter the atmosphere; reducing water pollution and decreasing soil erosion risks. These benefits are considered by consumer in their purchasing decision. For example, the protection of biodiversity is viewed as one of the most important additional ethical attributes of organic food for consumer purchasing decisions [57]. Gadema and Oglethorpe [58] examined whether carbon foot printing and labeling food products as a tool would actively facilitate consumers to make ‘greener’ purchasing decisions. They found that the majority of consumers stated preferences to carbon labels. Moreover, consumers believe that organic products are also good for soil, compared to conventional products [18]. The use of environmentally friendly packaging labels is also an environmental concern for many European consumers with regard to organic foods [59].

A number of research groups have also shown that trust is an important determinant of consumer preferences toward organic foods and food safety [21,29,60,61]. Since consumer motivations to purchase organic foods are influenced by a consumer’s trust in the logo/label, a higher trust in the logo determines a consumer’s willingness to pay for organic products [20]. Moreover, consumer distrust in the authenticity of goods is an issue that reduces consumer preferences toward organic foods in Thailand since they are not sure that the foods are genuinely organic [32]. Equally important is trusting the certification process. Krystallis and Chryssohoidis [62] found that the WTP a premium for organic foods depends on trust in the organic food certification process, and in the sellers of organic foods. In addition, Essoussi and Zahaf [63] explained that distribution, certification, country of origin, and labeling are all related to consumers’ level of trust when consuming organic foods. Drawing from the studies mentioned above, this study was conducted in order to define the determinants of consumers’ purchase behavior of organic vegetables, i.e., the attitudes towards organic foods, safety, and health, as well as environmental concerns and trust in organic attributes.

Socio-demographic characteristics may also influence consumer choices on organic food consumption. According to Stolz et al. [24], higher income is significantly related to a consumer’s preference to purchase organic foods and conventional plus (product categorized as ‘in between’ organic and conventional products, or food products with particular attributes that also apply to organic products). Similarly, income has been found to be a factor that explains consumer preferences in purchasing organic foods in some empirical studies [22,31,60]. Gracia and Magistris [64] indicated that income has an impact on organic food choice in the south of Italy, as lower-income consumers are less likely to buy organic foods. Consumers with higher incomes are also more likely to consume safe vegetables in Vietnam [35]. Hoang and Nakayasu [31] also showed that younger and well-educated consumers are more likely to buy safe vegetables. Meanwhile, females with children under 18 years of age, and small household size, are indicated as a significant factors in explaining consumer choices on organic product in Reference [65]. Many studies [24,31,42,60] pointed out that price is also a crucial factor for consumers purchasing organic foods. Therefore, socio-demographic variables, i.e., age, gender, educational level, household size, income, employment status of women, and having children younger than 18 years old, were investigated in this study and then incorporated with factors driving consumers, such as attitudes toward organic foods, safety and health concern, environmental concern, and trust in organic attributes.

## 3. Materials and Methods 

In this study, a total of 887 interviews were completed among household respondents of aged 18 years and older. Data were collected during February–March 2015 through door-to-door interviews by trained interviewers. The interviewer gathered the information in a home visit in order to encourage a high level of cooperation and complete responses. The surveys were conducted throughout the Jabodetabek region by dividing the region into four zones, i.e., Jakarta and Depok City; Bogor City and Bogor Regency; Tangerang City and South of Tangerang City; and Bekasi City and Bekasi Regency. This region was selected as the research area due to its high concentration of supermarkets and vegetable specialty stores that provide organic vegetables. According to Suharjo et al. [3], organic marketing in Indonesia is commonly based on these distribution channels (supermarkets and specialized organic stores). The present study was focused on the household sampling unit. Based on the report by Statistics Indonesia 2015 (see Table 1), the number of households in the research area is 7,178,528. A convenience sampling method was used to select the respondents. The distribution of questionnaires was based on the representative proportion of the household number in each zone in the Jabodetabek region.

The questionnaire used in the interviews were divided into two sections. The first section covered respondents’ socio-demographic characteristics (e.g., age, gender, education, family size, income, status of employed women in the household, and children under 18 years of age in the household). In the second section, collected data were aimed at understanding consumers’ purchase behavior between organic and conventionally grown vegetable and respondents’ opinions about factors that drive the purchasing organic vegetables. We interviewed respondents who have indicated that have heard the term ‘organic’, and asked whether they had ever purchased any organic vegetables in the past. Furthermore, respondents were asked to answer on the importance/agreement of statements related to driving factors in purchasing organic vegetables. The statements were selected with respect to attitudes toward organic foods, environmental issues, safety, health concerns, and trust, as discussed in the previous section. The attitude toward organic foods was measured using five belief statements: “How strongly do you agree or disagree with the statement that organic food is … than conventionally grown?” (1) higher in quality; (2) healthier; (3) safer; (4) fresher; and (5) tastier. The answers were given on 7-point scale, ranging from strongly disagree (1) to strongly agree (7). Most of the items were adopted from previous studies [18,47,52,69]. Next, the degree of consumers’ environmental concerns was measured by asking for the importance of (1) low carbon emissions; (2) balance and efficient in resource uses; (3) environmentally friendly packaging; and (4) environmentally friendly cultivation, answered on a 7-point Likert scale, ranging from ‘‘very unimportant” (1) to ‘‘very important” (7). Using the same Likert scale, consumers were also asked on motives related to safety and health concerns using the following statements: (1) I prefer to buy vegetables that have met the chemical residues standard; (2) It is important to purchase vegetables that are free from pathogenic microorganisms; (3) I prefer to buy vegetables cultivated using organic pesticides, herbicides and fertilizers; and (4) I consume vegetables to keep me and my family healthy, using a scale ranging from “strongly disagree” (1) to “strongly agree” (7). The statements related to environmental, safety, and health motives were adapted from food choice questionnaires derived from Steptoe et al. [70], and used in previous studies [48]. Consumer trust regarding organic attributes was also measured using a 7-point Likert using statements adapted from Voon et al. [30]: (1) “I trust the outlet that sells organic vegetable”; (2) “If the product is labeled and/or certified as organic, I trust it to be genuinely organic”; (3) “I believe that the product is really organic”, where 1 = strongly disagree and 7 = strongly agree. Consumer also asked about price “How do you perceive price of organic vegetables compared to conventionally grown vegetables?”, then chose 1 (expensive) or 0 (not expensive).

Descriptive analyses were conducted in order to show a respondent’s socio-demographic variables, followed by an analysis of the two-stage process, i.e., factor analysis and binary logistic regression. First, factor analysis was applied using the principle component analysis (PCA) method as a variable reduction procedure. The large number of different driving factors of consumers made it impossible to use each one as an explanatory variable in the subsequent analysis. For this reason, the analysis was conducted using summarized variables with the help of PCA. To ensure the suitability of conducting a factor analysis, this study used the Kaiser–Mayer–Olkin (KMO) test and Bartlett’s test of sphericity. The KMO test measures the adequacy of a sample in terms of the distribution of values in order to execute the factor analysis; the acceptable values should be greater than 0.5. Bartlett’s test of sphericity determines whether the correlation matrix is an identity matrix. Furthermore, to ensure the reliability of each factor, Cronbach’s coefficient alpha was also applied to test the internal consistency among the items included in each factor. The reliability refers to the consistency of the results for different items in the test [71].

Second, the contribution of obtained variables to main factors was tested using the binary logistic regression model, which was based on the obtained data of the developed model. For the binary response variable *y*, it denotes two categories: 1 and 0 [72]. In this paper, consumer choices to purchase organic vegetables is *y* = 1, when preferring organic vegetables; and otherwise, *y* = 0. Assuming that the probability of *y* = 1 is *P*; the function of *y* is as follows:

(1) $$f\left(y\right)=\;P^{y}{\left(1-P\right)}^{1-y},\;y=0,\;1$$

This paper utilizes the maximum likelihood estimation method to compute the regression parameter. The logit model’s basic form is as follows:

(2) $$P_{i}=F(\alpha +\;\overset{m}{\sum }\beta_{j}X_{ij}+\;u)=1/(1+Exp[-(\alpha +\overset{m}{\sum }\beta_{j}X_{ij}+\;u)])$$

where *Pi* is the probability of *I* (the serial number of a consumer), β_*j*_ is the regression parameter of influencing factors, *j* is the serial number of influencing factors, *m* is the number of influencing factors, *X_ij_* is the independent variable representing influencing factor *j* in sample *i*, α is the intercept, and *u* is the error. In the case in which *P*, which is strictly between 0 and 1, the method is simply to transform *P* and to obtain Logit (*y*) = ln [*P/*(1 − *P*)]. Logit, the log of the odds ratio, is not only linear in *X*, but is also, from the estimation view point, linear in the parameters. The relationship among purchase behavior, socio-demographic variables, and factor loading is concluded as follows: consumer’s purchase behavior of organic food = *F* (age, employed women, education, income, factor loadings, etc.) + random disturbing factor. The logistic model formula and independent variables are described below:

(3) $$Logit\;\left(y\right)=ln\;(\;\frac{P_{i}}{1-\;P_{i}})=\;\alpha +\beta_{1}X_{1}+\beta_{2}X_{2}+\beta_{3}X_{3}+\beta_{4}X_{4}+\beta_{5}X_{5}+\beta_{6}X_{6}+\beta_{7}X_{7}+\;\beta_{n}X_{n}+\;u$$

where y is the consumer’s purchase behavior of vegetables (1 = organic vegetables purchased in the past at least once, 0 = never purchased organic vegetables in the past); *X*_1_ is the consumer’s age; *X*_2_ is gender; *X*_3_ the consumer’s educational level; *X*_4_ is the consumer’s income level; *X*_5_ is the consumer’s employed status as a women in the household; *X*_6_ is children in the household; *X*_7_ is household size; and *X*_n_ is the consumer’s driving factors in organic vegetable purchasing in factor scores obtained from a factor analysis. The purpose of factor scores is to overcome multicollinearity problems in regression analysis [73].

## 4. Results

### 4.1. Socio-Demographic Profile of the Respondents

The socio-demographic characteristics of consumers are shown in Table 2. To analyze differences between consumer types, the respondents were asked questions regarding the vegetable purchasing behavior for their household. The respondents were divided into two groups: (a) those who had purchased any organic vegetable (organic group: 337 respondents, i.e., 38%); and (b) those who had never purchased any organic vegetable in the past (conventionally grown group: 550 respondents, i.e., 62%). In terms of age groups, 23% of total respondents were in the group of 18 to 25 years, 27% were in the group of 26 to 35 years, 37% were in the group of 36 to 50 years, and 13% were older than 51 years old. For respondents who chose conventionally grown vegetables, the ages present an older group (more than 35 years old); while for respondents who chose organic vegetables, the ages vary between 18 and 35 years old. The proportion of women respondents in the sample was higher than male respondents. This result conforms to the results of a previous study in Indonesia, which indicated that women tend to make a majority of the household purchase decisions [74]. Further, the data also show that respondents who chose conventionally grown vegetables typically had completed secondary school (almost 50%). While respondents who chose organic vegetables show a higher educational level, more than 52% had completed university. The results of chi-square tests revealed a significant difference in education levels between the organic and conventionally grown respondent group (χ^2^ = 30.257, *p*-value = 0.01).

Most respondents who chose conventionally grown vegetables have four or more family members (family households), while most respondents who chose organic vegetables have four or less family members. This difference is significant and supported by the chi-square results (χ^2^ = 12.370, *p*-value < 0.01). Likewise, in both groups, the majority of the respondents reported having children younger than 18 years in the household. The rate of employed women in the households was determined to be 40% from total respondents and differed significantly between the two groups (χ^2^ = 7.291, *p*-value < 0.01): 45% in the organic respondent group and almost 37% in the conventionally grown the respondent group. The income level of respondents noted mostly less than IDR 7,000,000 per month. However, the share of the organic respondent group that reported more than IDR 7,000,000 monthly income (12.8%) is significantly greater (χ^2^ = 5.404, *p*-value < 0.10) than organically grown respondent group (8.2%). Most respondents in both groups stated that the prices of organic products are more expensive than conventionally grown products.

### 4.2. Factors Driving Organic Vegetable Purchasing 

**Figure 1:**
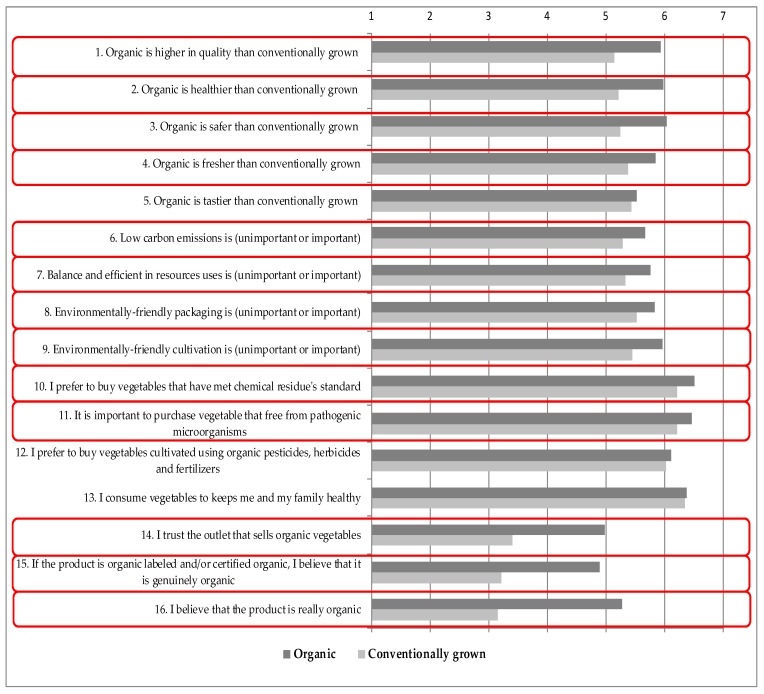
Arithmetic mean values of consumers’ evaluations of statements of the factors driving organic vegetable purchasing; statements with significant differences at the 1% level, based on *t*-test analysis between both groups, are framed.

There were questions based on a 7-point Likert scale with which respondents conveyed their opinions regarding factors driving their vegetable purchasing behavior. The differences in the statement evaluation between both consumer groups are presented in Figure 1. The highest mean value was found for the items on safety and health (statements 2, 3, 10, 11, 12, 13, and 14). This value indicates that both respondent groups believe that organic foods are indeed better for consumers’ health, compared to conventionally grown products. Moreover, they perceived safety and health to be more important aspects of vegetable purchasing. The lowest mean value was obtained for items related to the trust aspects (statements 14, 15 and 16), which asked how consumers perceived their beliefs toward organic stores, organic labels/certificates, and the originality of organic products. Organic consumers seem to have a higher degree of agreement with all the statements compared to conventionally grown consumers. Therefore, a differences analysis (based on *t*-test analysis, *p*-value < 0.05) was carried out in order to investigate possible significant mean differences between the groups. Significant differences between organic and conventionally grown consumers were found in 13 out of 16 statements, except statements 5, 12, and 13, where both sets of consumer answers tended to be similar.

Before the logistic analysis, the contribution of the explanatory variables to the consumers’ motives factor was obtained from the PCA. All loadings below 0.5 were dropped, and the factor analysis then re-calculated. Cronbach’s alpha was used to measure internal reliability by unit weighing items with salient loadings in a factor. The Kaiser-Meyer-Olkin (KMO) test of sampling adequacy and Bartlett’s test of sphericity were primarily performed on all the statements in order to confirm the appropriateness of applying factor analysis. The result of the KMO test was that the value was 0.830 and showed that the sampling adequacy was adequate. Kaiser [75] recommends values greater than 0.5 as being minimally acceptable and that values of 0.8 or above are meritorious [76].

The factor loading from the principal component of factor analysis was conducted after the varimax rotation. To obtain the rotated factor matrix, only items with a factor loading of 0.5 and above were considered valid items. Based on the results of the rotated factor matrix, the statements were grouped into their respective factors and were named according to their collective representations, i.e., “attitude towards organic foods”, “environmental concern”, “safety and health concerns”, and “trust” (Table 3). These four factors were combined to explain 76.0 percent of the total variance. The factor loading for four factors was from 0.613 to 0.932. Cronbach’s alpha was applied to test the reliability of variables. The results of test category showed a clear coherence amongst each category, with the attitude towards organic factor possessing an alpha value of 0.919 (5 items), the environmental factor a value of 0.925 (4 items), safety and health possessing an alpha value of 0.799 (4 items), and the trust factor possessing an alpha value of 0.907 (3 items).

### 4.3. Determinants Influencing Organic Purchasing 

The binary logistic model was used to estimate the determinants influencing organic vegetable purchasing. Table 4 shows the results of the model, including the coefficients (β), their standard errors (S.E.), associated *p*-values (Sig.), and odds ratio (Exp(β)). The final −2 Log Likelihood is 661.561, and decreased relative to the initial −2 Log Likelihood (1174.113). The Cox and Snell *R*^2^ is 0.440, which means that 44% of the variation in dependent variables is explained by the model. The Nagelkerke *R*^2^ was also estimated for the modification of Cox and Snell *R*^2^, and was found to be 0.598, which indicates that almost 60% of the variation in the dependent variable is explained by the model. The observed significance level of Chi-square value was found to be 0.610 (Hosmer and Lemeshow test), which indicates a rejection of the null hypothesis of the model, which means there is no difference between observed and predicted values. The model has the coefficient of predicting power of about 82.5%.

As shown in Table 4, consumers’ attitudes toward organic foods (ATTITUDE), consumers’ concerns for environmental issues (ENVIRONMENT), consumers’ concerns for their own health and safety (SAFETY & HEALTH), the degree of trust in organic vegetables (TRUST), degree of acceptance of current prices (PRICE), gender (GENDER), household size (HSIZE), and income above IDR 7,000,000 (INCOME3) have a statistically significant impact on the purchase behavior of organic vegetables. Meanwhile, age (AGE), educational level (EDU2 and EDU3), employment status of woman (EMPLOY), income between IDR 3,000,000–IDR 7,000,000 (INCOME2), and having children younger than 18 years (CHILD) in the household, have no significant impact on purchase behavior.

## 5. Discussion

The results of logistic model indicate that, as expected, the ATTITUDE, ENVIRONMENT, SAFETY & HEALTH, and TRUST variables have a positive effect on the purchasing of organic vegetables, which means that these variables are the main reasons that attract consumers to buy organic vegetables. In the case of consumers who have positive attitudes toward organic foods, they are more likely to purchase organic vegetables. Previous studies have demonstrated that the main factors affecting consumers’ purchasing decisions are mostly connected to their beliefs that organically grown products are healthier [43,50], better in quality [18], tastier [43], fresher [19], and safer [77] than conventional alternatives. Furthermore, since product development and marketing strategies are developed based on consumers’ beliefs and attitudes [78], extensive campaigns and communication in order to build up positive attitudes among consumers towards organic foods are needed. To improve the perceived utility of organic products, it is also important to integrate product-relevant information in communication strategies [23].

Safety and health motives (SAFETY & HEALTH) have a positive influence on consumers’ purchasing of organic vegetables. The common belief of consumers on organic products is that they have no pesticides, no artificial fertilizers, and are residue-free safe products [31]. These attributes were used to distinguish between organically and conventionally grown products. Consumers choices on organic vegetables depends on their perception of the nutritional value and health risks [39]. Many consumers often perceive organically grown food as healthier food with a higher content of vitamins and minerals, and are more environmental friendly compared to conventional products [42,43,44,55]. The expectation of good health as a main reason for purchasing organic food was similar with other studies [19,35,36]. The importance of health and safety in determining consumers’ purchasing decisions also confirms the results from most previous studies on organic foods that stated the reasons for Indonesian consumers in purchasing organic foods are safety and health concerns [3,12,16,79,80].

The ENVIRONMENT coefficient is statistically significant and positive. This coefficient indicates that consumers, who emphasized the importance of environmental attributes, are more likely to buy organic vegetables. This finding supports the empirical evidence of several studies [19,21,30,32,33,34,35,36] that indicate that environmental awareness determines consumers’ to purchase organic foods in many Southeast Asian countries. As stated in preceding studies [1,81], the establishment of organic agriculture in Indonesia was triggered by environmental issues as a response of the green revolution campaign. The application of synthetic pesticides, herbicides, and synthetic fertilizers to support high productivity has eventually led to environmental problems, such as declining soil organic matter and fertility, nutrient deficiency, soil and water pollution, erosion, and greenhouse effects [1]. Public awareness of organic agriculture had been low in past years, ao non-governmental organizations (NGOs) and the government of Indonesia made various efforts to promote the practice. Thus, the public has developed an interest in environmentally friendly organic farming [82]. Furthermore, based on our findings, promoting organic vegetables, i.e., information regarding how organic vegetable production and processing differ from conventional production, as well as how beneficial they are for the environment, should be emphasized. This will make potential consumers more willing to buy organic vegetables.

The positive estimated coefficient for the TRUST variable indicates that consumers’ trust in organic attributes is an important predictor of consumers’ purchasing of organic vegetables. In the organic food market, consumer trust is an important issue since consumers are unable to verify whether a product is an organic product. In determining the quality of a product, in addition to the search attributes (e.g., price, dimension, size, color) and experience attributes (e.g., taste, convenience, durability), there are also credential attributes. For search and experience attributes, consumers can evaluate prior to and after consumption; however, for credential attributes, consumers cannot ascertain directly at any stage of purchase, even after consumption of the food [83]. To solve the inability of consumers to evaluate credence attributes, and to gain their trust, these characteristics should be transformed into search attributes, which often shown on product labels as a certification logo from a third-party certification body [84,85]. Trust in labeling and certification has also been found to be an important motivator for Thai [21] and Malaysian consumers [36] in their decision to purchase organic vegetables.

In terms of socio-demographic variables, the GENDER variable has negative coefficient, which indicates that female respondents are less likely to purchase organic vegetables. This result is different to reports in the literature [15,65]. However, this is similar to the work by Roitner-Schobesberger et al. [19] on Thailand’s consumers, which found that men seem to be more likely purchase organic foods than women. The INCOME2 variable positively affects consumers’ choices to buy organic vegetables instead of conventionally grown vegetables. This implies that consumers with incomes above IDR 7,000,000 are more likely to buy organic vegetables. This result is confirmed by References [19,22,31,35,39,60] which also found that consumers with high levels of income are more likely to buy organic food products. However, it is in contrast with results from previous studies [65] that did not find any correlation between income and organic food buying choice. Furthermore, the negative coefficient of the HSIZE variable indicates that larger families are less likely to buy organic vegetables. This result is in line with Stolz et al. [24] and Loureiro et al. [65], who found that an increasing household size may reduce the probability of choosing organic products. However, this finding is in contrast with References [22,39,40] that did not find a significance of the household size variable in determining consumers choices. Finally, we also found that the PRICE variable was the major obstacle in buying organic foods for most consumers, which is consistent with previous studies [14,19,26,86]. This means that higher prices for organic vegetables, compared to conventionally grown vegetables, is a relevant factor for not purchasing organic vegetables.

## 6. Limitations and Conclusions 

### 6.1. Limitations

The limitations of this study pertain to the use of non-probability sampling. The use of such a sampling method and respondent selection procedure biased the sample towards consumers who have higher education; hence, generalizations that encompass the overall population need to be further validated. Furthermore, the findings from this research indicate that consumers’ organic vegetable purchasing behavior is influenced by their positive attitude towards organic foods (healthier, better in quality, tastier, fresher, and safer) and their concerns for health, safety, and the environment. However, whether organic foods actually delivers on these beliefs is still the subject of scientifically debate [87]. Therefore, in order to assess the possible bias resulting from this study, the positive attitude of consumers should be validated by their knowledge of organic food and organic farming methods. Consumer beliefs that may influence their attitude and behavior towards organic vegetable purchasing could also be distinguished as objective (accurate information about the product) or subjective (perceptions of what or how much they know about a product that may or may not be accurate) [88]. For further research, it is recommended that the influence of consumers’ beliefs about organic food together with the factors investigated in this study are examined using more representative consumer samples for all socio-demographic characteristics.

### 6.2. Conclusions

Results from this paper are of great importance because they provide valuable information on consumers’ purchase behavior in Indonesia, which can be used for organic vegetable marketing strategies. Several previous studies from many researchers have been conducted in order to analyze consumers’ organic food preferences in different regions in Indonesia over the last few years. Our current results are extending the knowledge of factors influencing Indonesian consumers’ organic vegetable purchasing behavior. The four most important factors were identified: attitude towards organic foods, environmental concerns, safety and health, as well as degree of trust. These factors are perceived by Indonesian consumers as motives in their purchasing of organic vegetables. Meanwhile, an interesting finding is that consumers’ socio-demographic characteristics indicate that only gender, household size, and income likely influence consumers in purchasing organic vegetables. Similar to other studies, price is still a crucial factor for consumers in deciding whether or not to purchase organic vegetables.

Furthermore, some recommendations for organic vegetable development in Indonesia can be derived from the findings in our study. First, it is necessary to have continuous communication in order to build awareness and perception of the benefits of organic vegetables. These benefits are often associated with positive attitudes toward organic foods, health, safety, and environmental sustainability. By emphasizing that organic vegetable production use fewer chemical inputs and meet food safety standards, consumers’ motives and concerns for health and safety can be addressed. Consumers should also be provided with appropriate and relevant information pertaining to organic production methods and the distinctiveness compared to conventional production methods, particularly regarding its impacts on the environment. Bai [89], based on experiences in Ehime Prefecture, Japan, illustrated that promoting the use of organic or eco-label farm products in school meals and developing farmers’ markets or increasing organic corners in supermarkets or food stores, will effectively promote the consumption of organic products and, secondly, that producers and retailers should implement pricing strategies for organic vegetables in order to increase organic vegetables purchases. According to Bezawada and Pauwels [90], lower regular prices would be effective for non-organic consumers or less-experienced organic consumers in stimulating purchases. However, Bunte et al. [91] noted that price elasticity for organic product is low, and lowering prices did not lead to an increase in product demand. On the other hand, price is viewed by consumers, not only as a cost, but also as a cue of quality; therefore, a medium or higher price level strategy could be implemented by producers and retailers as a pricing strategy [92]. This is because the price has an inverted U-shape relationship with the purchase of organic products, which means that organic demand will increase up to a certain point with higher prices, but, after this point, will demand decreases with increasing prices [93]. Third, in order to increase the degree of consumers’ trust towards organic vegetables, it is necessary to establish labeling and certification for products. The Ministry of Agriculture Regulation No. 64/Permentan/OT.140/5/2013 requires products labeled as ‘organic’ to be certified by an approved third party certification body, and include the logo “ORGANIC INDONESIA” on the packaging. According to Jahroh [81], the roles of a certification body are important in order to improve quality management systems of organic production, increase market demand, support initiation of internal control systems, especially in small farmer groups, and increase product image on the market. However, because the majority of organic producers in Indonesia are small-scale farmers, a great deal of such a regulation is not accessible, as they are forced to seek expensive third party certification [94]. Therefore, supporting and incentivizing small-scale farmers to access standardization and certification bodies is very important.

## Figures and Tables

**Table 1:** The number of households in the research area and the distribution of respondents.

Zone	Number of Household ^a^	Percentage of Household	Number of Respondents	Percentage of Respondents
Jakarta and Depok City	3,226,314	45%	384	43%
Bogor City and Bogor Regency	1,545,800	22%	240	27%
Tangerang City and South of Tangerang City	909,085	13%	126	14%
Bekasi City and Bekasi Regency	1,506,329	21%	137	15%
Total	7,187,528	100%	887	100%

^a^ Source: Badan Pusat Statistik (BPS)–Statistic Indonesia, 2015 [66,67,68].

**Table 2:** Socio-demographic characteristics of the respondents and variable definitions.

Characteristics (Variable Name in the Model)	Value Assignment in the Model	Mean and Std. dev ^a^	Number of Sample and (%)	Respondent Group (%)		Chi-Square	Sig. ^b^
				Conventionally Grown	Organic		
Number of respondents			887	550 (62%)	337 (38%)		
Age (AGE), years	Actual age	36.55 (11.815)				8.730	0.033
18–25			201 (22.7%)	20.2%	26.7%		
26–35			237 (26.9%)	26.9%	26.4%		
35–50			335 (37.8%)	38.0%	37.8%		
51+			114 (12.6%)	14.9%	9.5%		
Gender (GENDER)		0.88 (0.328)				0.571	0.450
Male	=0		109 (12.3%)	11.6%	13.4%		
Female	=1		778 (87.7%)	88.4%	86.6%		
Education level						30.527	0.000
Primary (9 years) or lower (EDU1)	=1, otherwise = 0		96 (10.8%)	14.2%	5.3%		
Secondary (EDU2)	=1, otherwise = 0		412 (46.6%)	49.3%	41.8%		
Tertiary or higher (EDU3)	=1, otherwise = 0		379 (42.6%)	35.5%	52.8%		
Household size (HSIZE)	Continuous	4.59 (2.27)				12.370	0.002
<2 people			95 (10.7%)	8.2%	14.8%		
2–4 people			331 (37.3%)	36.4%	38.9%		
>4 people			461 (52.0%)	55.5%	46.3%		
Children < 18 years (CHILD)		0.65 (0.478)				0.432	0.511
No	=0		312 (35.2%)	36.0%	33.8%		
Yes	=1		575 (64.8%)	64.0%	66.2%		
Employment status of women (EMPLOY)		0.40 (0.490)				7.291	0.007
Not employed	=0		532 (59.9%)	63.5%	54.3%		
Employed	=1		335 (40.1%)	36.5%	45.7%		
Monthly income in IDR 000 ^c^						5.404	0.067
<3000 (INCOME1)	=1, otherwise = 0		429 (48.4%)	50.2%	45.4%		
3000–7000 (INCOME2)	=1, otherwise = 0		370 (41.8%)	41.6%	41.8%		
>7000 (INCOME3)	=1, otherwise = 0		88 (9.8%)	8.2%	12.8%		
Organic price (PRICE)		0.89 (0.312)				0.226	0.634
Not expensive	=0		97 (10.9%)	10.5%	11.6%		
Expensive	=1		790 (89.1%)	89.5%	88.4%		

^a^ Arithmetic means and their respective standard deviations, ^b^ Significant, ^c^ 1 USD = IDR 13,100.

**Table 3:** Summary of factor analysis result.

Factors	Factor Loadings	Cronbach’s Alpha	Variance Explained (%)
**F1**: **Attitude towards organic foods (ATTITUDE)**		0.919	23.7
Organic is higher in quality than conventionally grown	0.906		
Organic is healthier than conventionally grown	0.884		
Organic is safer than conventionally grown	0.859		
Organic is fresher than conventionally grown	0.856		
Organic is tastier than conventionally grown	0.827		
**F2**: **Environmental concern (ENVIRONMENT)**		0.925	20.2
Low carbon emissions is (unimportant or important)	0.897		
Balance and efficient in resources uses is (unimportant or important)	0.894		
Environmentally friendly packaging is (unimportant or important)	0.883		
Environmentally friendly cultivation is (unimportant or important)	0.817		
**F3**: **Safety and Health concern (SAFETY & HEALTH)**		0.799	16.1
I prefer to buy vegetables that have met chemical residue’s standard	0.854		
It is important to purchase vegetables that are free from pathogenic microorganisms	0.851		
I prefer to buy vegetables cultivated using organic pesticides, herbicides and fertilizers	0.718		
I consume vegetables to keeps me and my family healthy	0.613		
**F4**: **Trust (TRUST)**		0.907	16.0
I trust the outlet that sells organic vegetables	0.932		
If the product is labeled and/or certified as organic, I believe that it is genuinely organic	0.925		
I believe that the product is really organic	0.891		

*p* < 0.001; extraction method, principle component analysis; rotation method, varimax with Kaiser normalization, total variance is 76.0.

**Table 4:** Estimated logit model for consumers’ organic vegetable purchasing.

	*Β*	S.E.	Sig.	Exp(β)
ATTITUDE	1.175	0.138	0.000 ***	3.239
ENVIRONMENT	0.304	0.103	0.003 ***	1.355
SAFETY & HEALTH	0.218	0.107	0.041 **	1.243
TRUST	1.957	0.139	0.000 ***	7.079
PRICE	−1.059	0.325	0.001 ***	0.347
AGE	−0.010	0.010	0.294	0.990
GENDER	−0.685	0.327	0.036 **	0.504
HSIZE	−0.100	0.056	0.073 *	0.905
EDU2	0.373	0.394	0.345	1.451
EDU3	0.299	0.432	0.488	1.349
INCOME2	0.097	0.238	0.685	1.101
INCOME3	0.942	0.386	0.015 **	2.565
EMPLOY	0.306	0.226	0.175	1.358
CHILD	0.035	0.226	0.878	1.035
Constant	0.952	0.682	0.163	2.590
L_0_ = −2 Log Likelihood (initial)		1174.113		
L_1_ = −2 Log Likelihood (final)		661.561		
Cox and Snell R square		0.440		
Nagelkerke R Square		0.598		
Hosmer and Lemeshow Test		0.610		
Prediction accuracy		82.5%		

*** Significant at 1%; ** significant at 5%; and * significant at 10%.
